# Investigation of the Interaction of Herbal Ingredients Contained in Triphala Recipe Using Simplex Lattice Design: Chemical Analysis Point of View

**DOI:** 10.1155/2020/5104624

**Published:** 2020-08-04

**Authors:** Chaowalit Monton, Thaniya Wunnakup, Jirapornchai Suksaeree, Laksana Charoenchai, Natawat Chankana

**Affiliations:** ^1^Drug and Herbal Product Research and Development Center, College of Pharmacy, Rangsit University, Pathum Thani 12000, Thailand; ^2^Department of Pharmaceutical Chemistry, College of Pharmacy, Rangsit University, Pathum Thani 12000, Thailand; ^3^Sun Herb Thai Chinese Manufacturing, College of Pharmacy, Rangsit University, Pathum Thani 12000, Thailand

## Abstract

The aim of this work was to investigate the interaction of herbal ingredients contained in Triphala recipe (*Terminalia chebula*, *Terminalia bellirica*, and *Phyllanthus emblica* in equal proportion) using simplex lattice design. This work focused on chemical analysis of four phenolic compounds including gallic acid, corilagin, chebulagic acid, and chebulinic acid by validated high-performance liquid chromatography. The effect of the extraction technique (decoction vs. infusion) and gamma irradiation was also examined. The combination index was used as a tool for determination of interaction of the ingredients contained in the herbal recipe. Results showed that the extraction technique and gamma irradiation slightly altered the content of some phenolic compounds as well as the combination index. The positive interaction seems to be found at the equal proportion of the three plants. This work scientifically supported the suitable formula of the Triphala recipe in the traditional use.

## 1. Introduction

Triphala is an herbal formula composed of equal proportion of dried fruits of three plants: *Terminalia chebula* Retz. var. *chebula*, *Terminalia bellirica* (Gaertn.) Roxb., and *Phyllanthus emblica* L. It has been used for a long time in Ayurvedic medicines. Triphala possesses numerous activities, including adaptogenic, antibacterial, antidiabetic, anti-inflammation, antimutagenic, antineoplastic, antioxidant, appetite stimulation, chemoprotective, immunomodulating, laxative, prevention of dental caries, radioprotective, and reduction of hyperacidity [1]. Triphala contains high content of ascorbic acid and phenolic compounds, so it possesses good antioxidant activity [2]. Pawar et al. [3] reported the use of gallic acid, chebulagic acid, and chebulinic acid as standard markers for Triphala. Charoenchai et al. [4] report the high-performance liquid chromatography (HPLC)- mass spectrometry profiles of Triphala and modified Triphala recipes. The major active compounds of Triphala are ascorbic acid, gallic acid, corilagin, chebulagic acid, rutin, chebulinic acid, and quercetin. Varma et al. [5] also reported the chemical constituents, including gallic acid, ellagic acid, chebulinic acid, emblicanin A and B, and friedelin. The other phenolic compounds were also reported, i.e., chebulic acid, tannic acid, epicatechin, and syringic acid [2]. Phenolic compounds usually act as an antioxidant; the high content of phenolic compounds has a positive effect on antioxidant activity [6–9]. Several studies reveal that Triphala is an antioxidant-rich product so that it can be used as a food supplement and beverage to promote health [10].

Synergistic effects or positive interaction of drugs or herbal plants enhance therapeutic effects or biological activities by some positive interactions among their different components [11]. The synergism is reported among herbal plants in numerous studies [12–19]. In some reports, herbal extract, as well as herbal plant, is synergized with some modern medicines, especially, essential oils with antibiotic [20–22]. Among several traditional medicines, traditional Chinese medicines are the most frequently reported in the literature to exhibit synergism of herbal plants [23, 24]. A synergistic effect in traditional Chinese medicines is reviewed. Multicomponent combinations of herbal plants are usually used in traditional medicines to boost the therapeutic efficacy and reduce side effects. The synergism can occur among herbs or other components in the prescription, among effective parts of herbs, and among bioactive compounds of herbs [25].

**Figure 1:**
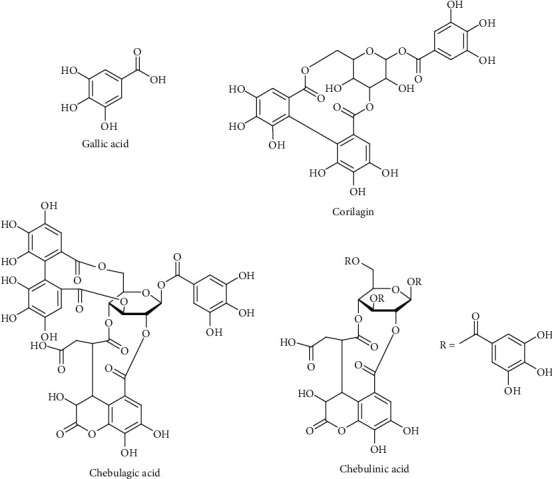
Chemical structures of phenolic compounds (gallic acid, corilagin, chebulagic acid, and chebulinic acid).

Triphala was previously reported for synergism with gentamicin or oxacillin against multidrug resistant gram-negative bacilli or multidrug resistant *Staphylococcus aureus*, respectively [26]. However, there is no report accounting for the synergism among the plant ingredients containing in its formula. So the aim of this work was to investigate the interaction of herbal ingredients contained in the Triphala recipe using simplex lattice experimental design. The 3D response surface analysis was applied to provide a complete description of the combination effect [27]. This work focused on the chemical analysis of four phenolic compounds, including gallic acid, corilagin, chebulagic acid, and chebulinic acid by using validated HPLC. Chemical structures of these phenolic compounds are shown in Figure 1. The effects of the extraction technique (decoction vs. infusion) and gamma irradiation (irradiation vs. nonirradiation) were also studied. The combination index was used as a tool for the determination of the interaction of the ingredients contained in the Triphala recipe. The authors expected that the data obtained from this work could be used to support the ratio of herbal ingredients contained in the Triphala recipe in traditional use.

## 2. Materials and Methods

### 2.1. Materials

The four reference standards, i.e., gallic acid (purity 99.88%), corilagin (purity 99.67%), chebulagic acid (purity 99.62%), and chebulinic acid (purity 98.42%), were purchased from Chengdu Biopurify Phytochemicals Ltd., China. The solvents were analytical and HPLC grades.

### 2.2. Preparation of Plant Samples

Dried fruits of *T. chebula*, *T. bellirica*, and *P. emblica* were purchased from Charoensuk Osod, Nakhon Pathom Province, Thailand. They were authenticated by Ajarn Nirun Vipunngeun, plant taxonomist and lecturer at Department of Pharmacognosy, College of Pharmacy, Rangsit University. *T. chebula*, *T. bellirica*, and *P. emblica* were deposited at the Drug and Herbal Product Research and Development Center, College of Pharmacy, Rangsit University. The voucher specimens were coded as CM-TC001-1-04-2019, CM-TB001-1-04-2019, and CM-PE001-1-04-2019, respectively. Seeds were removed from the fruits of each plant. The obtained fruits were pulverized using a grinder and stored in a dry place until use.

### 2.3. Simplex Lattice Experimental Design for Extraction of Plant Samples and the Optimization

The simplex lattice design was applied in this work. The weight fraction of *T. chebula*, *T. bellirica*, and *P. emblica* ranged from 0 to 1, and the summation of the weight fraction of the three plants was equal to 1. The independent variables of this work were the proportion of *T. chebula* (*X*_1_), *T. bellirica* (*X*_2_), and *P. emblica* (*X*_3_). The 12 formulas of plant mixture were prepared: F1 to F9 contained *T. chebula*, *T. bellirica*, and *P. emblica* in proportions of 1 : 0 : 0, 0 : 1 : 0, 0 : 0 : 1, 0.5 : 0.5 : 0, 0.5 : 0 : 0.5, 0 : 0.5 : 0.5, 0.66 : 0.17 : 0.17, 0.17 : 0.66 : 0.17, and 0.17 : 0.17 : 0.66, respectively. F10 to F12 were the same formula. They contained equal proportion of *T. chebula*, *T. bellirica*, and *P. emblica* (0.33 : 0.33 : 0.33).

The plant powder mixture was used for extraction by decoction and infusion methods. In case of decoction, plant powder mixture (6 g) was placed in a tea bag and boiled in water (50 mL) for 15 min. The tea bag with powder mixture was removed and boiled again three times in total. The three parts of the obtained solution were pooled and filtered through Whatman® filter paper no.1. It was lyophilized using a freeze dryer for 18-24 hours. The lyophilized extract powder was collected and kept in desiccator until use.

In case of infusion, plant powder mixture (6 g) was placed in a tea bag, placed in boiling water (50 mL), and stood for 15 min. The tea bag with powder mixture was removed and infused again three times in total. The three parts of the obtained solution were pooled and filtered. It was lyophilized similar to the decoction group. The lyophilized extract powder was collected and kept in desiccator until use.

The extract powder obtained from decoction was sampled to gamma irradiation (10 kGy). The three groups of samples, i.e., extract from decoction without gamma irradiation, extract from decoction with gamma irradiation, and extract from infusion, were obtained. They have analyzed for content of phenolic compounds, including gallic acid, corilagin, chebulagic acid, and chebulinic acid. The six dependent variables, i.e., extraction yield (*Y*_1_), gallic acid content (*Y*_2_), corilagin content (*Y*_3_), chebulagic acid content (*Y*_4_), chebulinic acid content (*Y*_5_), and total content of the four phenolic compounds (*Y*_6_) were monitored. They were used to produce the 3D response surface by Design-Expert® version 11.0. Furthermore, the equations for prediction of each dependent factor were created.

### 2.4. Analysis of Phenolic Compounds

The HPLC was used to analyze the four phenolic compounds, including gallic acid, corilagin, chebulagic acid, and chebulinic acid. The aqueous solutions of the extract of F1 to F12 were prepared in a concentration of 2 mg/mL, except F3 which was prepared in a concentration of 0.5 mg/mL, and F6 and F9 were prepared in a concentration of 1 mg/mL. The content of the four phenolic compounds was calculated based on the calibration curve of each compound.

The analysis of phenolic compounds was performed using an HPLC instrument (Agilent 1260 infinity, Agilent, USA). The ACE C18-PFP column (250 × 4.6 mm, internal diameter, 5 *μ*m) was used. The column temperature was controlled at 25°C. The mobile phase was composed of acetonitrile (A) and 1% acetic acid aqueous solution (B). The gradient elution system was similar to the previous work of Charoenchai et al. [4]. It was started by holding 5% A for 1 min, increased to 10% A in 3 min, increased to 15% A in 8 min, increased to 35% A in 20 min, increased to 50% A in 3 min, increased to 100% A in 2 min and holding for 3 min, and decreased to 5% A in 1 min and holding for 4 min. The mobile phase flow rate was 1 mL/min. The injection volume was 10 *μ*L. The photodiode array detector was set at 270 nm.

This method was validated to confirm its linearity, range, the limit of detection (LOD) and limit of quantitation (LOQ), specificity, precision, and accuracy. The linear equations and coefficient of determination (*R*^2^) values of calibration curves of gallic acid, corilagin, chebulagic acid, and chebulinic acid in the test range of 10-200 *μ*g/mL were *y* = 4039955*x* + 6926719 (*R*^2^ = 0.9991), *y* = 2292681*x* + 9273157 (*R*^2^ = 0.9978), *y* = 1672107*x* + 197458 (*R*^2^ = 1.0000), and *y* = 2068580*x*–298060 (*R*^2^ = 0.9999), respectively. The LOD and LOQ values were 2.50 and 7.57 *μ*g/mL, 0.46 and 1.39 *μ*g/mL, 0.98 and 2.96 *μ*g/mL, and 0.84 and 2.55 *μ*g/mL, respectively. The percent relative standard deviations of intraday precision and interday precision of all phenolic compound standards were lower than 2% and 5%, respectively. The accuracy values of the analysis of gallic acid, corilagin, chebulagic acid, and chebulinic acid were 95.23-105.2%, 91.74-107.0%, 99.22-104.3%, and 92.98-101.2%, respectively. Furthermore, this method was specifically due to the UV spectrums of the individual peak of each phenolic compound in the extract similar to the UV spectrum of each standard compounds.

### 2.5. Investigation of Interaction

The interaction was investigated based on the response additivity approach, which is also referred to as the linear interaction effect. The posotove interaction occurred when the observed combination effect was higher than the expected additive effect given by the summation of the individual effect [27]. However, this work focused on the content of phenolic compounds, so the positive interaction in this work occurred when the observed content of phenolic compounds from the combination plants was higher than the expected additive content given by the summation of the individual plant. The tool used to investigate the interaction was adapted from the combination index (CI), a practical model used for determination of synergism of a multicomponent mixture in a fixed ratio [24], as

(1) $$\begin{array}{c} \text{CI}=\frac{E_{A}+E_{B}+E_{C}}{E_{\text{combination}}}, \end{array}$$

where *E*_*A*_, *E*_*B*_, *E*_*C*_ were the individual effect of *T. chebula*, *T. bellirica*, and *P. emblica*, respectively. *E*_combination_ was the observed combination effect of *T. chebula*, *T. bellirica*, and *P. emblica*. The positive interaction, additive effect, and negative interaction occurred when the CI values were lower than 1, equal to 1, and higher than 1, respectively [24, 27].

The response surface methodology was also applied to clarify the interaction. The six dependent variables of CI values of the extraction yield (*Y*_7_), gallic acid content (*Y*_8_), corilagin content (*Y*_9_), chebulagic acid content (*Y*_10_), chebulinic acid content (*Y*_11_), and total content of the four phenolic compounds (*Y*_12_) were monitored. They were used to produce the contour plots by Design-Expert® version 11.0. The equations for the prediction of each dependent variable were created. The plots between the predicted values and the actual values were produced, and the *R*^2^ values were reported to explain the level of correlation. The plots between internally studentized residuals and the run numbers were also produced to demonstrate the level of the distribution of the data. The contour plots of the desirability of the optimal condition provided the simultaneous minimizing CI of the extraction yield (*Y*_7_), and total content of the four phenolic compounds (*Y*_12_) was created. Finally, the overlay plots that CI values of both extraction yield and total content of four phenolic compounds of less than 0.5 and 0.4 were reported.

## 3. Results and Discussion

### 3.1. Content of the Phenolic Compounds

**Figure 2:**
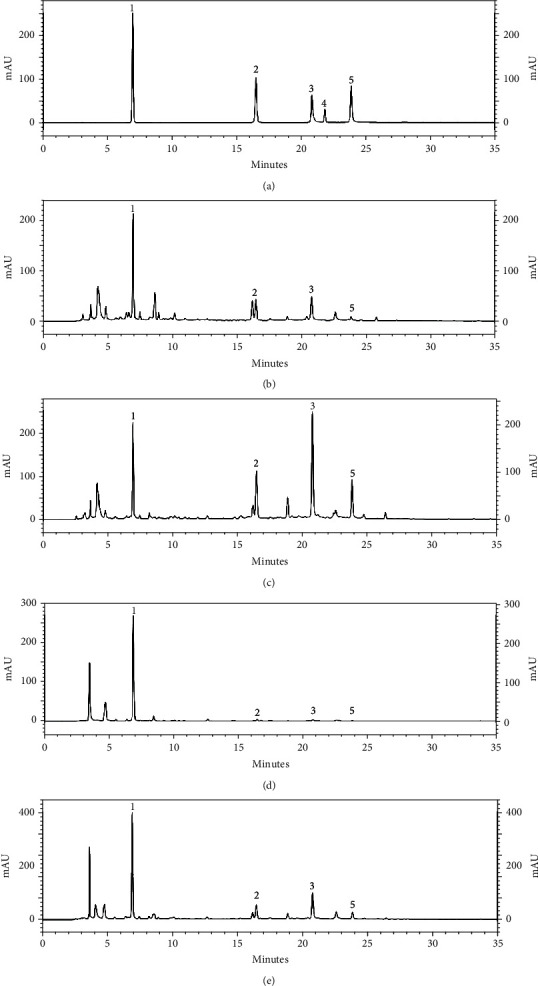
HPLC chromatograms of (a) mixed standards of gallic acid (1), corilagin (2), chebulagic acid (3), rutin (4), and chebulinic acid (5) in each concentration of 50 *μ*g/mL, (b) F1 decoction extract (2 mg/mL), (c) F2 decoction extract (2 mg/mL), (d) F3 decoction extract (0.5 mg/mL), and (e) F10 decoction extract (2 mg/mL).

Charoenchai et al. [4] reported that chemical constituents contained in Triphala obtained from electrospray mass spectrometry were ascorbic acid, gallic acid, corilagin, chebulagic acid, rutin, chebulinic acid, and quercetin (small amount). The five phenolic compounds, including gallic acid, corilagin, chebulagic acid, rutin, and chebulinic acid were selected as standard markers for Triphala used in this study. The previous work demonstrated that HPLC could be used as an important instrument for quantitation of chemical constituents in the Triphala recipe. The HPLC chromatograms of mixed standards and the formulas are shown in Figure 2. The highest chemical compound found in *T. chebula* and *P. emblica* was gallic acid, while the highest chemical compound of *T. bellirica* was chebulagic acid. However, rutin was not found in all 12 formulas. So only gallic acid, corilagin, chebulagic acid, and chebulinic acid were further quantified.

The mathematic equations used to predict each response (*Y*_1_ to *Y*_6_) are shown in Equations (2)-(19). Among the proportion of *T. chebula* (*X*_1_), *T. bellirica* (*X*_2_), and *P. emblica* (*X*_3_), the *X*_2_ had the highest effect on the extraction yield (*Y*_1_), corilagin content (*Y*_3_), chebulagic acid content (*Y*_4_), chebulinic acid content (*Y*_5_), and total content of the four phenolic compounds (*Y*_6_) of all groups, except, *Y*_1_ of the infusion group was affected the greatest by the *X*_1_. According to the gallic acid content (*Y*_2_) of all groups, they were the greatest affected by *X*_3_.

#### 3.1.1. Decoction without Gamma Irradiation

(2) $$\begin{array}{c} Y_{1}=17.56X_{1}+20.89X_{2}+16.38X_{3}+90.24X_{1}X_{2}+67.88X_{1}X_{3}+67.88X_{2}X_{3}+114.00X_{1}X_{2}X_{3}, \end{array}$$

(3) $$\begin{array}{c} Y_{2}=0.29X_{1}+0.40X_{2}+1.47X_{3}+2.32X_{1}X_{2}+2.81X_{1}X_{3}+4.97X_{2}X_{3}, \end{array}$$

(4) $$\begin{array}{c} Y_{3}=0.10X_{1}+0.49X_{2}+0.08X_{3}+1.48X_{1}X_{2}+0.21X_{1}X_{3}+2.05X_{2}X_{3}-3.81X_{1}X_{2}X_{3}, \end{array}$$

(5) $$\begin{array}{c} Y_{4}=0.28X_{1}+1.78X_{2}+0.29X_{3}+3.53X_{1}X_{2}+0.02X_{1}X_{3}+5.90X_{2}X_{3}, \end{array}$$

(6) $$\begin{array}{c} Y_{5}=0.03X_{1}+0.56X_{2}+0.06X_{3}+0.89X_{1}X_{2}-0.31X_{1}X_{3}+1.55X_{2}X_{3}, \end{array}$$

(7) $$\begin{array}{c} Y_{6}=0.73X_{1}+3.24X_{2}+1.92X_{3}+7.89X_{1}X_{2}+2.42X_{1}X_{3}+14.14X_{2}X_{3}. \end{array}$$

#### 3.1.2. Decoction with Gamma Irradiation

(8) $$\begin{array}{c} Y_{1}=17.56X_{1}+20.89X_{2}+16.38X_{3}+90.24X_{1}X_{2}+67.88X_{1}X_{3}+67.88X_{2}X_{3}+114.00X_{1}X_{2}X_{3}, \end{array}$$

(9) $$\begin{array}{c} Y_{2}=0.32X_{1}+0.30X_{2}+0.99X_{3}+1.75X_{1}X_{2}+3.78X_{1}X_{3}+4.49X_{2}X_{3}, \end{array}$$

(10) $$\begin{array}{c} Y_{3}=0.10X_{1}+0.47X_{2}-0.02X_{3}+1.67X_{1}X_{2}+0.48X_{1}X_{3}+0.24X_{2}X_{3}, \end{array}$$

(11) $$\begin{array}{c} Y_{4}=0.48X_{1}+1.89X_{2}+0.01X_{3}+6.17X_{1}X_{2}+1.24X_{1}X_{3}+1.28X_{2}X_{3}, \end{array}$$

(12) $$\begin{array}{c} Y_{5}=0.29X_{1}+0.62X_{2}+0.01X_{3}+1.29X_{1}X_{2}-0.34X_{1}X_{3}+0.62X_{2}X_{3}, \end{array}$$

(13) $$\begin{array}{c} Y_{6}=1.19X_{1}+3.27X_{2}+1.00X_{3}+10.88X_{1}X_{2}+5.16X_{1}X_{3}+6.62X_{2}X_{3}. \end{array}$$

#### 3.1.3. Infusion

(14) $$\begin{array}{c} Y_{1}=21.29X_{1}+17.55X_{2}+11.98X_{3}+99.33X_{1}X_{2}+73.50X_{1}X_{3}+58.07X_{2}X_{3}, \end{array}$$

(15) $$\begin{array}{c} Y_{2}=0.24X_{1}+0.33X_{2}+1.06X_{3}+2.14X_{1}X_{2}+4.31X_{1}X_{3}+2.85X_{2}X_{3}, \end{array}$$

(16) $$\begin{array}{c} Y_{3}=0.05X_{1}+0.41X_{2}-0.01X_{3}+1.46X_{1}X_{2}+0.46X_{1}X_{3}+0.96X_{2}X_{3}, \end{array}$$

(17) $$\begin{array}{c} Y_{4}=0.24X_{1}+1.88X_{2}+0.001X_{3}+5.89X_{1}X_{2}+1.79X_{1}X_{3}+4.60X_{2}X_{3}, \end{array}$$

(18) $$\begin{array}{c} Y_{5}=0.02X_{1}+0.71X_{2}+0.05X_{3}+1.49X_{1}X_{2}+0.17X_{1}X_{3}+1.15X_{2}X_{3}, \end{array}$$

(19) $$\begin{array}{c} Y_{6}=0.55X_{1}+3.33X_{2}+1.11X_{3}+10.98X_{1}X_{2}+6.73X_{1}X_{3}+9.56X_{2}X_{3}, \end{array}$$

**Figure 3:**
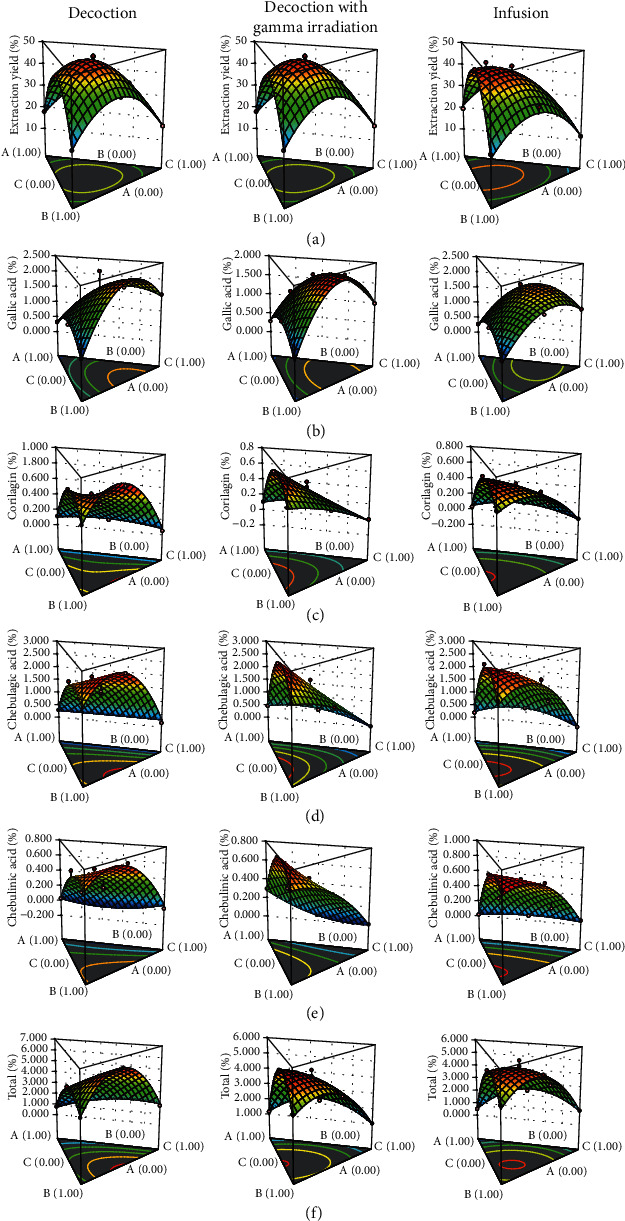
3D response surfaces of model conditions of (a) extraction yield, (b) gallic acid content, (c) corilagin content, (d) chebulagic acid content, (e) chebulinic acid content, and (f) total content of four phenolic compounds. *A*, *B*, and *C* were *T. chebula*, *T. bellirica*, and *P. emblica*, respectively.

Figure 3 shows the 3D response surfaces of model conditions of extraction yield, the individual content of phenolic compounds, and total content of four phenolic compounds. The high extraction yields of decoction groups with or without gamma irradiation and infusion group were found at the equal proportion of the three plants (Figure 3(a)). The extraction yields obtained from decoction and infusion were 16.33–49.33% and 11.83–45.50%, respectively. The high gallic acid content of the decoction group without gamma irradiation was achieved at the low proportion of *T. chebula* with a medium proportion of *T. bellirica* and medium to the high proportion of *P. emblica.* The high gallic acid content of the decoction group with gamma irradiation and the infusion group was achieved at the low to medium proportion of *T. chebula* with the low to medium proportion of *T. bellirica* and the medium to high proportion of *P. emblica* (Figure 3(b)). The high corilagin content of the decoction group without gamma irradiation was achieved at the low proportion of *T. chebula* with the medium proportion of *T. bellirica* and the medium proportion of *P. emblica.* The high corilagin content of the decoction group with gamma irradiation and the infusion group was achieved at the low to medium proportion of *T. chebula* with the medium to high proportion of *T. bellirica* and the low proportion of *P. emblica* (Figure 3(c)). The high chebulagic acid content of the decoction group without gamma irradiation was achieved at the low to medium proportion of *T. chebula* with the medium to high proportion of *T. bellirica* and the low to medium proportion of *P. emblica.* The high chebulagic acid content of the decoction group with gamma irradiation was achieved at the low to medium proportion of *T. chebula* with the medium to high proportion of *T. bellirica* and the low proportion of *P. emblica.* The high chebulagic acid content of the infusion group was achieved at the low to medium proportion of *T. chebula* with the medium to high proportion of *T. bellirica* and the low to medium proportion of *P. emblica* (Figure 3(d)). The high chebulinic acid content of the decoction group without gamma irradiation was achieved at the low to medium proportion of *T. chebula* with the medium to high proportion of *T. bellirica* and the low to medium proportion of *P. emblica.* The high chebulinic acid content of the decoction group with gamma irradiation was achieved at the low to medium proportion of *T. chebula* with the medium to high proportion of *T. bellirica* and the low proportion of *P. emblica.* The high chebulinic acid content of the infusion group was achieved at the low to medium proportion of *T. chebula* with the medium to high proportion of *T. bellirica* and the low to medium proportion of *P. emblica* (Figure 3(e)). The high total content of four phenolic compounds of the decoction group without gamma irradiation was achieved at the low to medium proportion of *T. chebula* with the medium proportion of *T. bellirica* and the medium proportion of *P. emblica.* The high total content of four phenolic compounds of the decoction group with gamma irradiation and the infusion group was achieved at the low to medium proportion of *T. chebula* with the medium to high proportion of *T. bellirica* and the low to medium proportion of *P. emblica* (Figure 3(f)). In addition, the 3D response surfaces revealed that gamma irradiation slightly altered the content as well as pattern of the response surfaces of some phenolic compounds. Decomposition of some unstable substances or alteration of some phenolic compounds by gamma irradiation was previously reported [28–31]. Furthermore, extraction techniques gave different phenolic compounds' content. The decoction seems to provide the higher extraction yield and content of phenolic compounds compared with the infusion. The author mentioned that infusion might be an alternative method of Triphala because it seems to be an energy-saving manner compared to decoction.

### 3.2. Interaction of Herbal Ingredients Contained in the Triphala Recipe

The mathematic equations used to predict each response (*Y*_7_ to *Y*_12_) are shown in Equations (20)-(37). Among the proportion of *T. chebula* (*X*_1_), *T. bellirica* (*X*_2_), and *P. emblica* (*X*_3_), all *X*_1_, *X*_2_, and *X*_3_ seem to have an equal effect on CI of the extraction yield (*Y*_7_), gallic acid content (*Y*_8_), corilagin content (*Y*_9_), chebulagic acid (*Y*_10_), chebulinic acid (*Y*_11_), and total content of the four phenolic compounds (*Y*_12_) of all groups. However, the interaction of the three plants significantly affected all dependent variables.

#### 3.2.1. Decoction without Gamma Irradiation

(20) $$\begin{array}{c} Y_{7}=0.99X_{1}+0.99X_{2}+0.98X_{3}-2.05X_{1}X_{2}-1.90X_{1}X_{3}-1.81X_{2}X_{3}, \end{array}$$

(21) $$\begin{array}{c} Y_{8}=0.98X_{1}+0.99X_{2}+1.01X_{3}-2.10X_{1}X_{2}-1.45X_{1}X_{3}-2.03X_{2}X_{3}, \end{array}$$

(22) $$\begin{array}{c} Y_{9}=0.98X_{1}+1.00X_{2}+0.93X_{3}-1.88X_{1}X_{2}-1.21X_{1}X_{3}-2.36X_{2}X_{3}, \end{array}$$

(23) $$\begin{array}{c} Y_{10}=0.98X_{1}+1.01X_{2}+0.93X_{3}-1.82X_{1}X_{2}-1.31X_{1}X_{3}-2.38X_{2}X_{3}, \end{array}$$

(24) $$\begin{array}{c} Y_{11}=0.98X_{1}+1.02X_{2}+0.91X_{3}-1.89X_{1}X_{2}-0.57X_{1}X_{3}-2.42X_{2}X_{3}, \end{array}$$

(25) $$\begin{array}{c} Y_{12}=0.98X_{1}+1.00X_{2}+0.96X_{3}-1.92X_{1}X_{2}-1.37X_{1}X_{3}-2.24X_{2}X_{3}. \end{array}$$

#### 3.2.2. Decoction with Gamma Irradiation

(26) $$\begin{array}{c} Y_{7}=0.99X_{1}+0.99X_{2}+0.98X_{3}-2.05X_{1}X_{2}-1.90X_{1}X_{3}-1.81X_{2}X_{3}, \end{array}$$

(27) $$\begin{array}{c} Y_{8}=0.96X_{1}+1.00X_{2}+0.99X_{3}-2.01X_{1}X_{2}-2.15X_{1}X_{3}-2.20X_{2}X_{3}, \end{array}$$

(28) $$\begin{array}{c} Y_{9}=0.99X_{1}+1.01X_{2}+1.00X_{3}-2.28X_{1}X_{2}-2.56X_{1}X_{3}-0.51X_{2}X_{3}, \end{array}$$

(29) $$\begin{array}{c} Y_{10}=0.97X_{1}+1.02X_{2}+0.97X_{3}-2.17X_{1}X_{2}-2.06X_{1}X_{3}-0.87X_{2}X_{3}, \end{array}$$

(30) $$\begin{array}{c} Y_{11}=0.99X_{1}+0.99X_{2}+0.99X_{3}-1.68X_{1}X_{2}+5.85X_{1}X_{3}-1.29X_{2}X_{3}-35.69X_{1}^{2}X_{2}X_{3}+18.20X_{1}X_{2}^{2}X_{3}-41.06X_{1}X_{2}X_{3}^{2}, \end{array}$$

(31) $$\begin{array}{c} Y_{12}=1.00X_{1}+1.00X_{2}+1.00X_{3}-2.15X_{1}X_{2}-2.04X_{1}X_{3}-1.68X_{2}X_{3}-6.19X_{1}^{2}X_{2}X_{3}+13.91X_{1}X_{2}^{2}X_{3}-2.67X_{1}X_{2}X_{3}^{2}. \end{array}$$

#### 3.2.3. Infusion

(32) $$\begin{array}{c} Y_{7}=1.00X_{1}+1.00X_{2}+1.00X_{3}-2.29X_{1}X_{2}-2.16X_{1}X_{3}-2.05X_{2}X_{3}-3.66X_{1}^{2}X_{2}X_{3}+14.24X_{1}X_{2}^{2}X_{3}-1.44X_{1}X_{2}X_{3}^{2}, \end{array}$$

(33) $$\begin{array}{c} Y_{8}=0.97X_{1}+1.00X_{2}+1.00X_{3}-2.14X_{1}X_{2}-2.26X_{1}X_{3}-1.65X_{2}X_{3}, \end{array}$$

(34) $$\begin{array}{c} Y_{9}=1.00X_{1}+1.00X_{2}+1.00X_{3}-2.48X_{1}X_{2}-3.54X_{1}X_{3}-2.22X_{2}X_{3}-6.96X_{1}^{2}X_{2}X_{3}+25.33X_{1}X_{2}^{2}X_{3}+0.68X_{1}X_{2}X_{3}^{2}, \end{array}$$

(35) $$\begin{array}{c} Y_{10}=1.00X_{1}+1.00X_{2}+1.00X_{3}-2.35X_{1}X_{2}-3.34X_{1}X_{3}-2.23X_{2}X_{3}-5.36X_{1}^{2}X_{2}X_{3}+26.86X_{1}X_{2}^{2}X_{3}-3.68X_{1}X_{2}X_{3}^{2}, \end{array}$$

(36) $$\begin{array}{c} Y_{11}=1.01X_{1}+1.01X_{2}+1.01X_{3}-1.92X_{1}X_{2}-1.40X_{1}X_{3}-1.63X_{2}X_{3}-21.43X_{1}^{2}X_{2}X_{3}+22.80X_{1}X_{2}^{2}X_{3}-0.58X_{1}X_{2}X_{3}^{2}, \end{array}$$

(37) $$\begin{array}{c} Y_{12}=1.00X_{1}+1.00X_{2}+1.00X_{3}-2.28X_{1}X_{2}-2.61X_{1}X_{3}-2.02X_{2}X_{3}-9.78X_{1}^{2}X_{2}X_{3}+20.65X_{1}X_{2}^{2}X_{3}-1.11X_{1}X_{2}X_{3}^{2}. \end{array}$$

**Figure 4:**
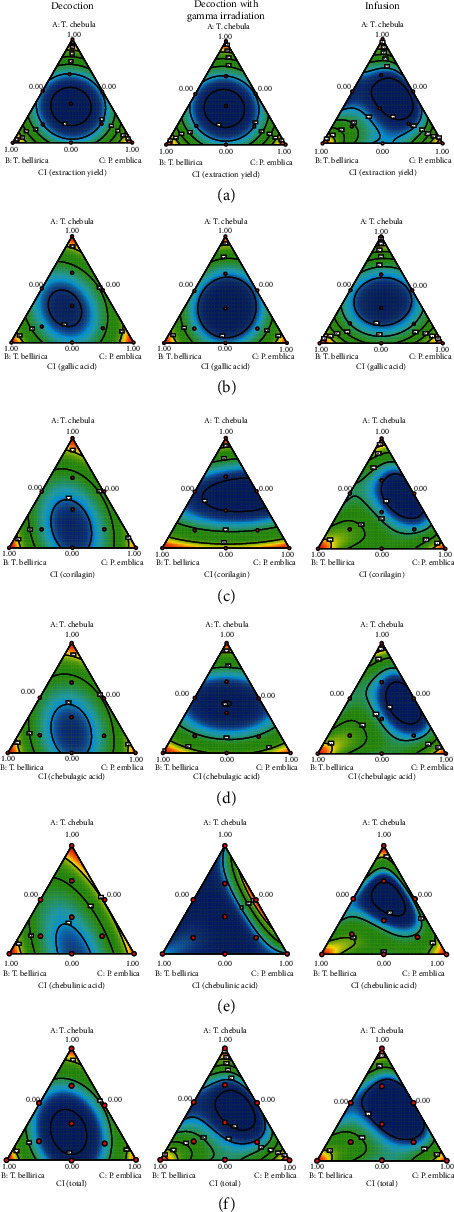
Contour plots of model conditions of combination index of (a) extraction yield, (b) gallic acid content, (c) corilagin content, (d) chebulagic acid content, (e) chebulinic acid content, and (f) total content of four phenolic compounds.

Figure 4 shows the contour plots of model conditions of CI of extraction yield, the individual content of phenolic compounds, and total content of four phenolic compounds. It was focused on the low CI value which reflects a high level of interaction. The low CI of the extraction yield of decoction groups with or without gamma irradiation was found at the equal proportion of the three plants. In case of the infusion group, the low extraction yield was found at the medium proportion of *T. chebula* with the low to medium proportion of *T. bellirica* and the medium proportion of *P. emblica* (Figure 4(a)). The low CI of gallic acid content of decoction groups with or without gamma irradiation and the infusion group were achieved at the equal proportion of the three plants (Figure 4(b)). The low CI of corilagin and chebulagic acid content of the decoction group without gamma irradiation was achieved at the low to medium proportion of *T. chebula* with the medium proportion of *T. bellirica* and the medium proportion of *P. emblica.* The low CI of corilagin and chebulagic acid content of the decoction group with gamma irradiation was achieved at the equal proportion of the three plants. The low CI of corilagin and chebulagic acid content of the infusion group was achieved at the medium proportion of *T. chebula* with the low proportion of *T. bellirica* and the medium proportion of *P. emblica* (Figures 4(c) and 4(d)). The low CI of chebulinic acid content of the decoction group without gamma irradiation was achieved at the low proportion of *T. chebula* with the medium proportion of *T. bellirica* and the medium proportion of *P. emblica.* The low CI of chebulinic acid content of the decoction group with gamma irradiation was achieved at the low to high proportion of *T. chebula* with the medium to high proportion of *T. bellirica* and the low to high proportion of *P. emblica.* The low CI of chebulinic acid content of the infusion group was achieved at the medium proportion of *T. chebula* with the low to medium proportion of *T. bellirica* and the medium proportion of *P. emblica* (Figure 4(e)). The low CI of total content of four phenolic compounds of the decoction group without gamma irradiation was achieved at the equal proportion of the three plants. The low CI of total content of four phenolic compounds of the decoction group with gamma irradiation and the infusion group was achieved at the medium proportion of *T. chebula* with the low to medium proportion of *T. bellirica* and the medium proportion of *P. emblica* (Figure 4(f)).

**Figure 5:**
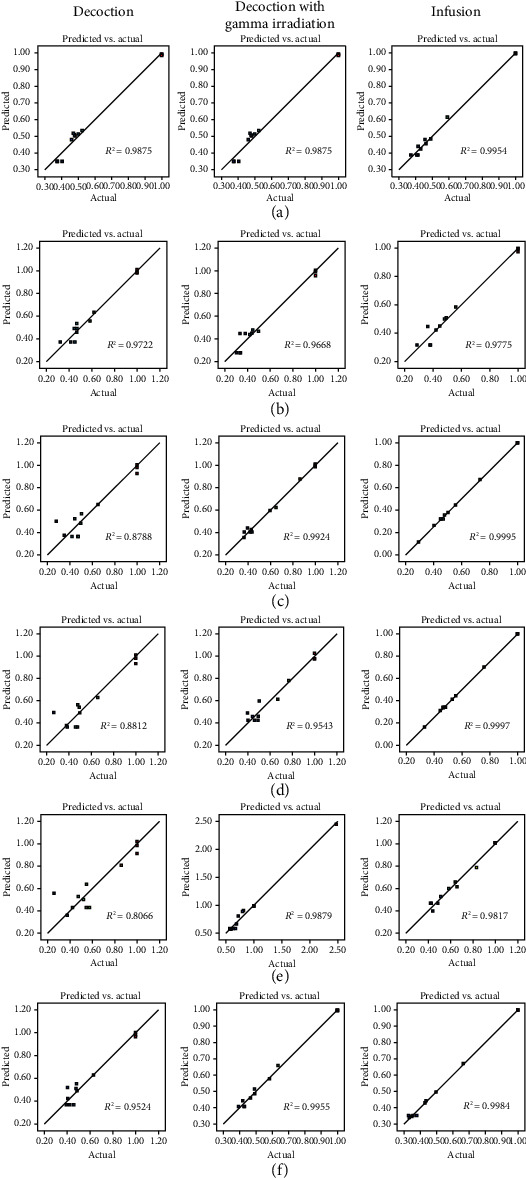
Predicted vs. actual value plots of model conditions of (a) extraction yield, (b) gallic acid content, (c) corilagin content, (d) chebulagic acid content, (e) chebulinic acid content, and (f) total content of four phenolic compounds.

**Figure 6:**
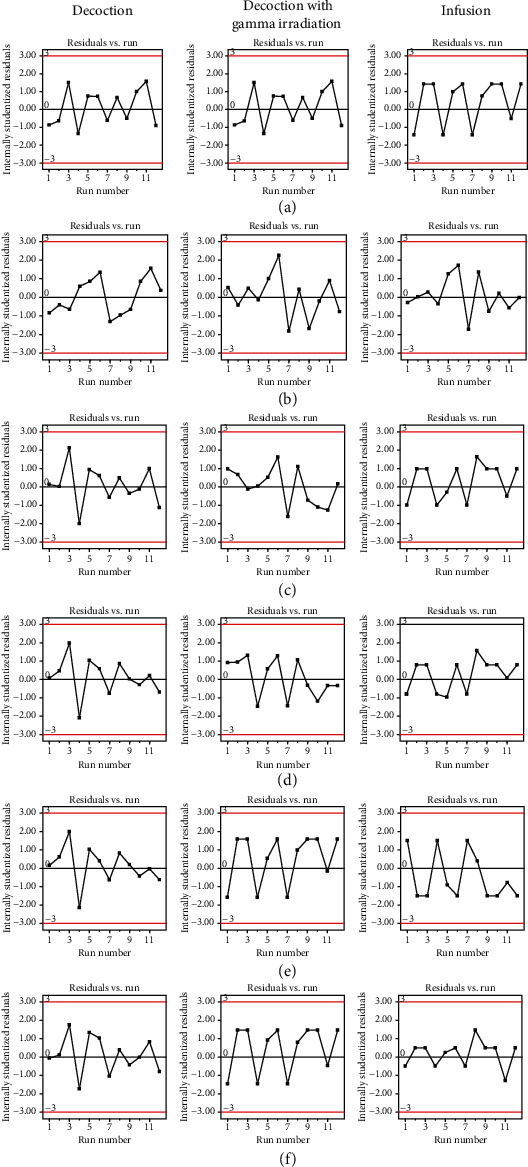
Residuals vs. run plots of model conditions of (a) extraction yield, (b) gallic acid content, (c) corilagin content, (d) chebulagic acid content, (e) chebulinic acid content, and (f) total content of four phenolic compounds.

The correlation plots between predicted vs. actual values of model conditions of CI of extraction yield, gallic acid content, corilagin content, chebulagic acid content, chebulinic acid content, and total content of four phenolic compounds are shown in Figure 5. Relative high *R*^2^ values indicated that the prediction of the Design-Expert® software was precise and reliable. The correlation plots between internally studentized residuals vs. run numbers of model conditions of CI of extraction yield, gallic acid content, corilagin content, chebulagic acid, chebulinic acid, and total content of four phenolic compounds are shown in Figure 6. All data were distributed within the limit (red line). They indicated that the data distribution was within 95% confident interval. It could be confirmed that the prediction by Design-Expert® software was stable [32–36].

**Figure 7:**
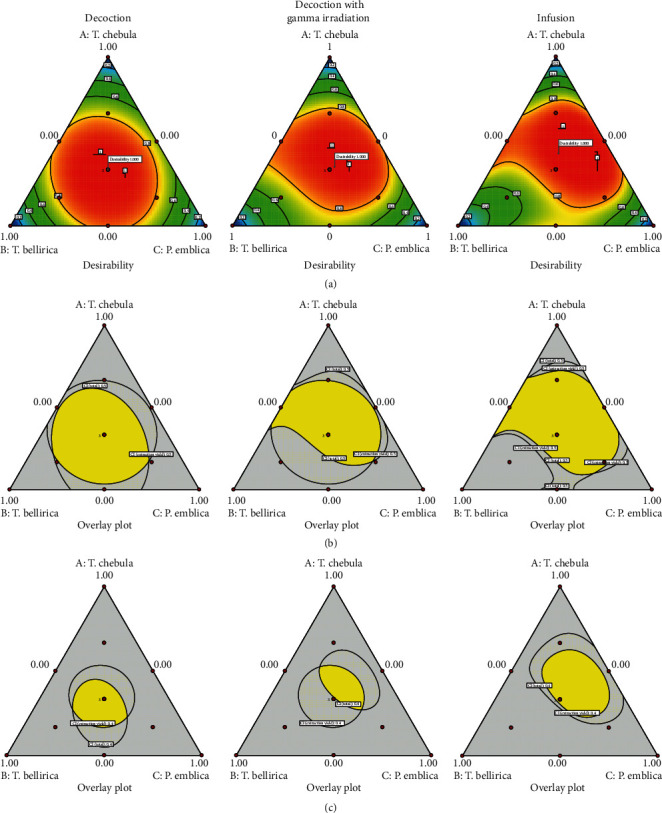
Desirability plots (a) of the optimal condition provided the simultaneous minimizing combination index of the extraction yield and total content of the four phenolic compounds and overlay plots (b, c) of the combination index of the extraction yield and total content of four phenolic compounds which the combination indices of both factors were lower than (b) 0.5 and (c) 0.4.

Figure 7(a) shows that contour plots of the desirability of the optimal condition provided the simultaneous minimizing CI of the extraction yield and total content of the four phenolic compounds. The maximized synergistic effect was found at the proportion of *T. chebula*, *T. bellirica*, and *P. emblica* of 0.33 : 0.33 : 0.33, 0.35 : 0.30 : 0.35, and 0.43 : 0.27 : 0.30 for the decoction without gamma irradiation group, the decoction with gamma irradiation group, and the infusion group, respectively. Figures 7(b) and 7(c) show the overlay plots of CI of extraction yield and total content of four phenolic compounds for which the values were less than 0.5 and 0.4, respectively. The yellow area of the overlay plots indicated the proportion of *T. chebula*, *T. bellirica*, and *P. emblica* providing the CI values of extraction yield and total content of four phenolic compounds of less than 0.5 (Figure 7(b)) or less than 0.4 (Figure 7(c)). According to the equal proportion of the three plants, the traditional Triphala recipe, the CI values were less than 0.5. It was indicated that the combination of equal proportion of *T. chebula*, *T. bellirica*, and *P. emblica* could promote the combination effect greater than the expected additive effect given by the summation of the individual effect for at least 200%. Because of the broad yellow area of overlay plots, the synergism could occur in a wide range of plant proportions apart from the equal ratio. When focused on the CI values of less than 0.4, the traditional Triphala recipe still promoted the combination effect greater than the expected additive effect for at least 250%. It could be established that the traditional Triphala recipe of decoction without gamma irradiation and infusion groups exhibited the positive interaction of more than 2.5 times of the individual effect of the single plant while the decoction group with gamma irradiation exhibited the positive interaction of 2 to 2.5 times of the individual effect of the single plant.

The interrelationship of the herbs in the herbal recipe could occur in different ways: two or more herbs reinforcing each other called synergism, herbs strengthening the effect of another herb called assisting, herbs reducing the curative effect of another herb called antagonism, and herbs increasing toxicity of another herb called rejection [37]. The synergism occurred among herbs or other components in the prescription, among effective parts of herbs, or among bioactive compounds of herbs [25]. In the case of the Triphala recipe, the positive interaction could be promoted by the herbal ingredients contained in the recipe or among active compounds of the recipe, which was investigated using validated HPLC combined 3D response surface analysis. The authors suggested that the positive interaction could be used as a tool to describe the mechanism of the synergism of the herbal formula. This data scientifically proved that the traditional Triphala recipe was appropriate to use due to their positive interaction based on chemical analysis point of view.

## 4. Conclusions

The investigation of the interaction of the three herbal ingredients contained in the Triphala recipe was designed based on the simplex lattice experimental design. The four phenolic compounds were selected as a chemical marker for determination of the interaction based on chemical analysis point of view. Results showed that the extraction techniques affected the extraction yield as well as content of phenolic compounds. The decoction gave more extraction yield and phenolic compound content compared with infusion. In addition, gamma irradiation could slightly alter the content of phenolic compounds. According to the interaction of extraction yield and total content of phenolic compounds, the positive interaction seems to be found at the equal proportion of *T. chebula*, *T. bellirica*, and *P. emblica*, which was similar to the traditional use. It was found that at those ratios, the extraction yield and total content of phenolic compounds increased at least two times the individual effect of each plant. In summary, the data of this work supported that the equal proportion of *T. chebula*, *T. bellirica*, and *P. emblica* in the Triphala recipe was already appropriate.

## Data Availability

The data used to support the findings of this study are available from the corresponding author upon request.
